# ^68^Ga-PSMA-11 PET imaging in patients with ongoing androgen deprivation therapy for advanced prostate cancer

**DOI:** 10.1007/s12149-021-01646-z

**Published:** 2021-06-29

**Authors:** Saskia Fassbind, Daniela A. Ferraro, Jean-Jacques Stelmes, Christian D. Fankhauser, Matthias Guckenberger, Philipp A. Kaufmann, Daniel Eberli, Irene A. Burger, Benedikt Kranzbühler

**Affiliations:** 1 Department of Urology, University Hospital Zürich, University of Zürich, Zurich, Switzerland; 2 Department of Nuclear Medicine, University Hospital Zürich, University of Zürich, Rämistrasse 100, 8091 Zurich, Switzerland; 3 Department of Radiation Oncology, University Hospital Zürich, University of Zürich, Zurich, Switzerland

**Keywords:** Prostate cancer, Prostate-specific antigen, ^68^Ga-PSMA-11, Positron emission tomography, PSMA

## Abstract

**Purpose:**

Prostate-specific membrane antigen (PSMA) targeted positron emission tomography (PET) imaging significantly improved the detection of recurrent prostate cancer (PCa). However, the value of PSMA PET imaging in patients with advanced hormone-sensitive or hormone-resistant PCa is still largely unknown. The aim of this study was to analyze the detection rate and distribution of lesions using PSMA PET imaging in patients with advanced PCa and ongoing androgen deprivation therapy (ADT).

**Methods:**

A total of 84 patients diagnosed with hormone-sensitive or hormone-resistant PCa who underwent ^68^Ga-PSMA-11 PET/magnetic resonance imaging (MRI) or computer tomography (CT) under ongoing ADT were retrospectively analyzed. We assessed the detection of PSMA-positive lesions overall and for three PSA subgroups (0 to < 1 ng/mL, 1 to < 20 ng/mL and > 20 ng/mL). In addition, PSMA-positive findings were stratified by localization (prostatic fossa, pelvic, para-aortic, mediastinal/supraclavicular and axillary lymph nodes, bone lesions and visceral lesions) and hormone status (hormone-sensitive vs. hormone-resistant). Furthermore, we assessed how many patients would be classified as having oligometastatic disease (≤ 3 lesions) and theoretically qualify for metastasis-directed radiotherapy (MDRT) in a personalized patient management.

**Results:**

We detected PSMA-positive lesions in 94.0% (79 of 84) of all patients. In the three PSA subgroups detection rates of 85.2% (0 to < 1 ng/mL, *n* = 27), 97.3% (1 to < 20 ng/mL, *n* = 37) and 100% (> 20 ng/mL, *n* = 20) were observed, respectively. PSMA-positive visceral metastases were observed only in patients with a PSA > 1 ng/mL. Detection of PSMA-positive lesions did not significantly differ between patients with hormone-sensitive and hormone-resistant PCa. Oligometastatic PCa was detected in 19 of 84 patients (22.6%). Almost all patients, 94.7% (*n* = 18) would have been eligible for MDRT.

**Conclusions:**

In this study, we observed an overall very high detection rate of 94% using PSMA PET imaging in patients with advanced PCa and ongoing ADT. Even in a majority of patients with very low PSA values < 1 ng/ml PSMA-positive lesions were found.

## Introduction

Prostate cancer (PCa) is still the most frequently diagnosed cancer and second most common cause of cancer-related death in men worldwide [1]. Prostate-specific membrane antigen (PSMA) targeted positron emission tomography (PET) in combination with computerized tomography (CT) or magnetic resonance imaging (MRI) significantly improved the detection of primary and recurrent PCa [2, 3]. Therefore, current guidelines recommend PSMA PET imaging for the early detection of recurrent disease if the results will influence subsequent treatment decisions [4]. However, the performance of PSMA PET imaging for patients with advanced PCa is still largely unknown [5]. Given that the influence of androgen deprivation therapy (ADT) on PSMA expression is not fully understood with first clinical data suggesting a higher PSMA uptake on PET in men treated with ADT [6, 7], a separate analysis of PET detection in patients under ADT is needed. Indeed first results suggest a very high detection rate of PSMA PET imaging in hormone-resistant PCa patients previously diagnosed as non-metastatic by conventional imaging [8]. However, still little is known about therapeutic consequences of these PSMA PET results in the setting of hormone-resistant PCa.

In addition, advances in imaging techniques have led to a more frequent detection of low-volume oligometastatic PCa, a state of diseases which was first proposed in the 1990s by Hellman and Weichselbaum [9]. They described a concept of oligometastatic tumors representing an intermediate state between initially non-metastatic and widely disseminated [10]. Though, there is still no consensus on the exact definition of oligometastatic PCa and it remains unknown weather treatment of oligometastatic disease can improve patient survival or delay the further development of metastases [11]. Prospective data from a randomized phase II study showed improved ADT-free survival in patients treated with stereotactic metastasis-directed radiotherapy (MDRT) to low-volume metastatic cancer compared with surveillance [12].

The aim of this study was to analyze the detection rate and distribution of lesions using PSMA PET imaging in patients with advanced PCa and ongoing ADT. In addition, we retrospectively assessed how many patients on ADT would be classified as having oligometastatic disease and theoretically qualify for MDRT.

## Patients and methods

### Patients

We retrospectively analyzed 84 patients with advanced PCa under ongoing ADT who underwent ^68^Ga-PSMA-11 PET/MRI or CT between May 2016 and April 2018 at our department. Patient characteristics including prostate-specific antigen (PSA) level at time of diagnosis and at time of scan, primary tumor stage, Gleason Score, surgical margin status and primary therapy regime were retrieved from patient records. ^68^Ga-PSMA-11 PET imaging detection rate was analyzed overall and for three PSA subgroups (0 to < 1 ng/mL, 1 to < 20 ng/mL and > 20 ng/mL). The cut-offs were selected to separate subgroups with very low PSA where other imaging modalities (e.g. choline PET/CT) are known to have a low detection rate (20%), and those, where also conventional imaging starts to detect disease with a threshold of 20 ng/ml. In addition, ^68^Ga-PSMA-11 PET positive findings were stratified by localization (prostatic fossa, pelvic, para-aortic, mediastinal/supraclavicular and axillary lymph nodes, bone lesions and visceral lesions). The local ethics committee approved the study protocol (BASEC Nr. 2016-02230) and all patients have signed a general written informed consent.

### ^68^Ga-PSMA-11 PET imaging

All patients underwent a single injection of ^68^Ga-PSMA-11 (mean dose ± standard deviation, 131 ± 17.9 MBq, range 84–171 MBq). To reduce ^68^Ga-PSMA-11 activity in the bladder, ureters, and kidneys, furosemide was injected intravenously 30 min prior to the ^68^Ga- PSMA-11 injection (0.13 mg/kg), and patients were asked to void prior to the scan.

### ^68^Ga-PSMA-11 PET/MRI protocol

A clinical routine whole-body ^68^Ga-PSMA-11 PET/MRI was performed 60 min after injection on a hybrid scanner (SIGNA PET/MRI, GE Healthcare, Waukesha, WI, USA) used in previous studies at our department with the same protocol for prostate imaging as recently described [13]. In brief, six bed positions with 2–3 min acquisition time per bed position for the whole-body protocol, and additional specific sequences covering the pelvis, including a high resolution T1-weighted LAVA-FLEX sequence, T2-weighted fast recovery fast spin-echo sequence in at least two planes and DWI was performed.

### ^68^Ga-PSMA-11 PET/CT protocol

For patients who underwent ^68^Ga-PSMA-11 PET/CT, PET was performed with six bed positions with 2.5 min acquisition time per bed position and an attenuation CT scan was acquired on a Discovery VCT 690 PET/CT (GE Healthcare, Waukesha, WI, USA) or on a Discovery MI PET/CT (GE Healthcare, Waukesha, WI, USA) 60 min after injection with whole-body scan parameters as follows: tube voltage 140 kV, tube current with automated dose modulation with a maximum of 80 mA/slice, collimation 512 × 0.976, pitch 0.984:1, rotation time 0.5 s, coverage speed 78 mm/s, field of view (FOV) 50 cm, and images with a transverse pixel size of 0.976 and a slice thickness of 1.25 mm reconstructed in the axial plane.

### ^68^Ga-PSMA-11 PET image analysis

All ^68^Ga-PSMA-11 PET images were analyzed using an Advantage Workstation (Version 4.6 or 4.7, GE Healthcare). This enables the review of the PET and the CT or MR images side by side and in fused mode. A dual board-certified radiologist and nuclear medicine physician (IAB) analyzed all images, incorporating both the MRI or CT and PET information as well as all clinical information. For all lesions, maximum standardized uptake value (SUV_max_) and size were assessed. Only lesions with high suspicion for recurrence were considered positive: focal ^68^Ga-PSMA-11 uptake in the soft tissue of the prostatic fossa, lymph nodes with an SUV_max_ ≥ 3.5 and/or pathologically increased size (≥ 5 mm for perirectal nodes, ≥ 8 mm for iliac/retroperitoneal nodes, ≥ 1 cm for inguinal nodes), focal bone uptake with correlating bone marrow replacement on MRI, or high uptake or focal sclerosis on CT was considered suspicious [13]. Given that most published series suggest values of SUV_max_ between 2 and 3 as appropriate cut off values for lymph nodes [14, 15], we selected SUV_max_ ≥ 3 to minimize false positive interpretation of slightly PSMA-positive findings. In this study inter-reader agreement was not assessed, given that previous work already showed excellent correspondence [16–18].

### Retrospective evaluation of oligometastatic patients diagnosed by ^68^Ga-PSMA-11 PET imaging

Oligometastatic disease was defined as less or equal than 3 PSMA-positive lesions [12, 19]. Only bone and lymph node metastases were included. In a second step, all scans together with the patient’s records were reviewed by a board-certified radiation oncologist and evaluated if a MDRT would have been applicable in a personalized setting.

### Statistical analysis

Statistical analysis was performed using GraphPad Prism version 8 (GraphPad Software, Inc. La Jolla, USA) and SPSS (IBM SPSS Statistics Version 25 (IBM, Armonk, NY, USA). Data is either presented as median (interquartile range) or number (percent). A fisher’s exact test or a chi-square-test was used to compare detection rates between 2 and 3 groups. *p* values < 0.05 are considered statistically significant.

## Results

### Patient characteristics, ^68^Ga-PSMA-11 PET detection rate and distribution of metastases

**Fig. 1:**
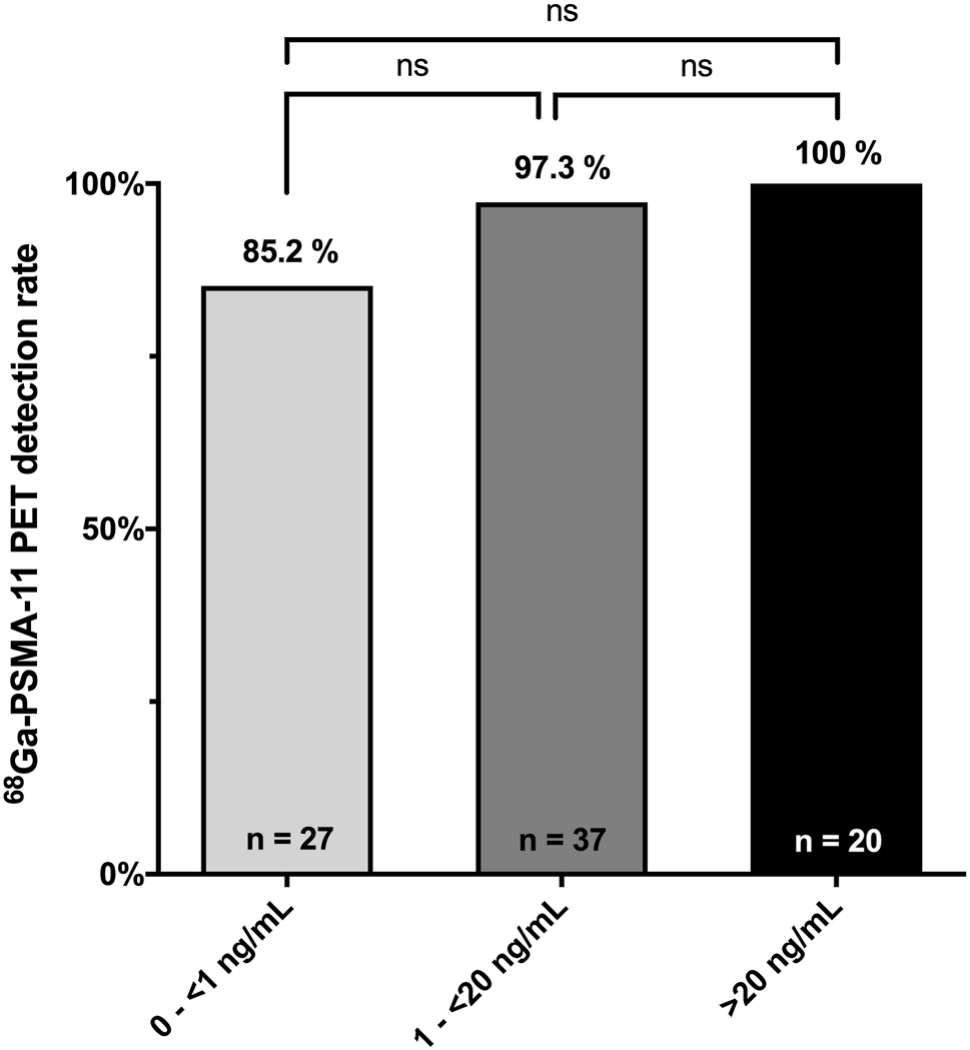
^68^Ga-PSMA-11 PET imaging detection rate stratified by three different PSA subgroups (0 to < 1 ng/mL, 1 to < 20 ng/mL and > 20 ng/mL) at time of scan. Data is shown as percentage of events. A fisher’s exact test was calculated; *p* values < 0.05 were considered statistically significant

We analyzed 84 patients with advanced hormone-sensitive or hormone-resistant PCa under ongoing ADT. Patient characteristics are summarized in Table 1. Overall, 30 (35.7%) patients with hormone-sensitive and 54 (64.3%) patients with hormone-resistant disease were included. Median time since start of ADT was 25 months (interquartile range IQR 10–49.5 months). The overall detection rate was 94.0%. In the three PSA subgroups a detection rate of 85.2% (0 to < 1 ng/mL, *n* = 27), 97.3% (1 to < 20 ng/mL, *n* = 37) and 100% (> 20 ng/mL, *n* = 20) was observed, respectively. No statistically different detection rates were observed between patients with low (0 to < 1 ng/mL), intermediate (1 to < 20 ng/mL; *p* = 0.15) and high (> 20 ng/mL; *p* = 0.12) PSA values at the time of scan (Fig. 1). All suspicious nodal lesion had a SUVmax of 3.5 or higher, suspicious local recurrence or bone metastasis had a minimum uptake of SUVmax 3.6, respectively.

**Table 1:** Patient characteristics

Characteristics	*n* = 84
Age at scan (years)	71 (66–76)
PSA (ng/ml)	
PSA at initial treatment	19.6 (9–66.5
PSA at scan time	4.27 (0.8–18)
Initial tumor stage (%)	
≤ T2c	27 (32.1)
≥ T3a	42 (50)
n/a, cTx	15 (17.9)
Initial lymph node stage (%)	
N0	33 (39.3)
N1	37 (44)
N2	1 (1.2)
n/a, cNx	13 (15.5)
Initial metastatic stage (%)	
M0	55 (65.5)
M1	20 (23.8)
n/a, cMx	9 (10.7)
Initial Gleason score (%)	
< 8	22 (26.2)
≥ 8	53 (63.1)
n/a	9 (10.7)
ADT at scan	
Duration of ADT (months)	25 (10–49.5)
Hormone status at scan (%)	
Hormone sensitive	30 (35.7)
Hormone resistant	54 (64.3)
Prior therapy (%)	
Prior prostate cancer-related surgery	43 (51.2)
Prior prostate cancer-related radiotherapy	41 (48.8)

Data presented as median (interquartile range) or number (percent)

*ADT* androgen deprivation therapy, *n/a* not available

**Fig. 2:**
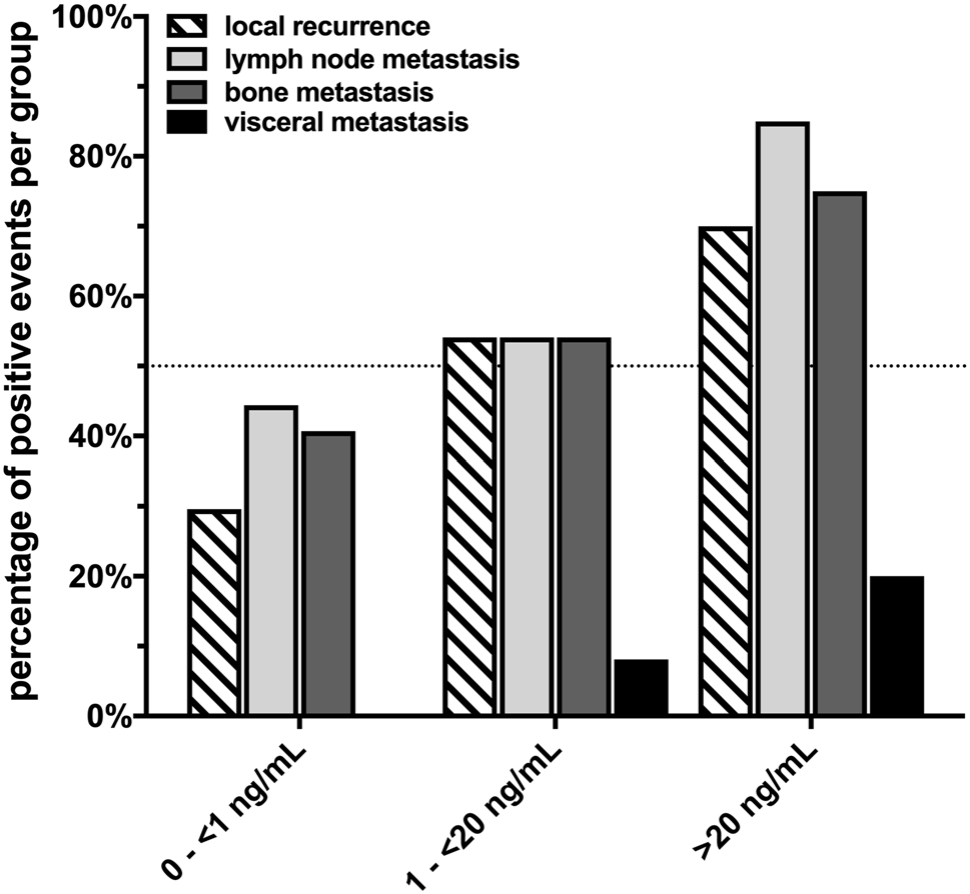
Number of ^68^Ga-PSMA-11 PET imaging positive events stratified by localization and PSA subgroups at time of scan. Data are shown as percentage of events

Distribution of suspicious lesions is shown in Fig. 2. In patients with PSA values 0 to < 1 ng/mL PSMA-positive lesions were found in the prostatic fossa in 29.6%, in lymph nodes in 44.4% and in bones 40.7%. No visceral metastases were recorded in this patient subgroup. Patients with PSA values 1 to < 20 ng/mL showed PSMA-positive lesions in the prostatic fossa in 54.1%, in lymph nodes in 54.1%, in bones 54.1% and visceral metastases in 8.1%. In patients with a PSA > 20 ng/mL at the time of scan corresponding PSMA-positive lesions were detected in the above-mentioned locations in 70.0%, 85.0%, 75% and 20%, respectively.

**Fig. 3:**
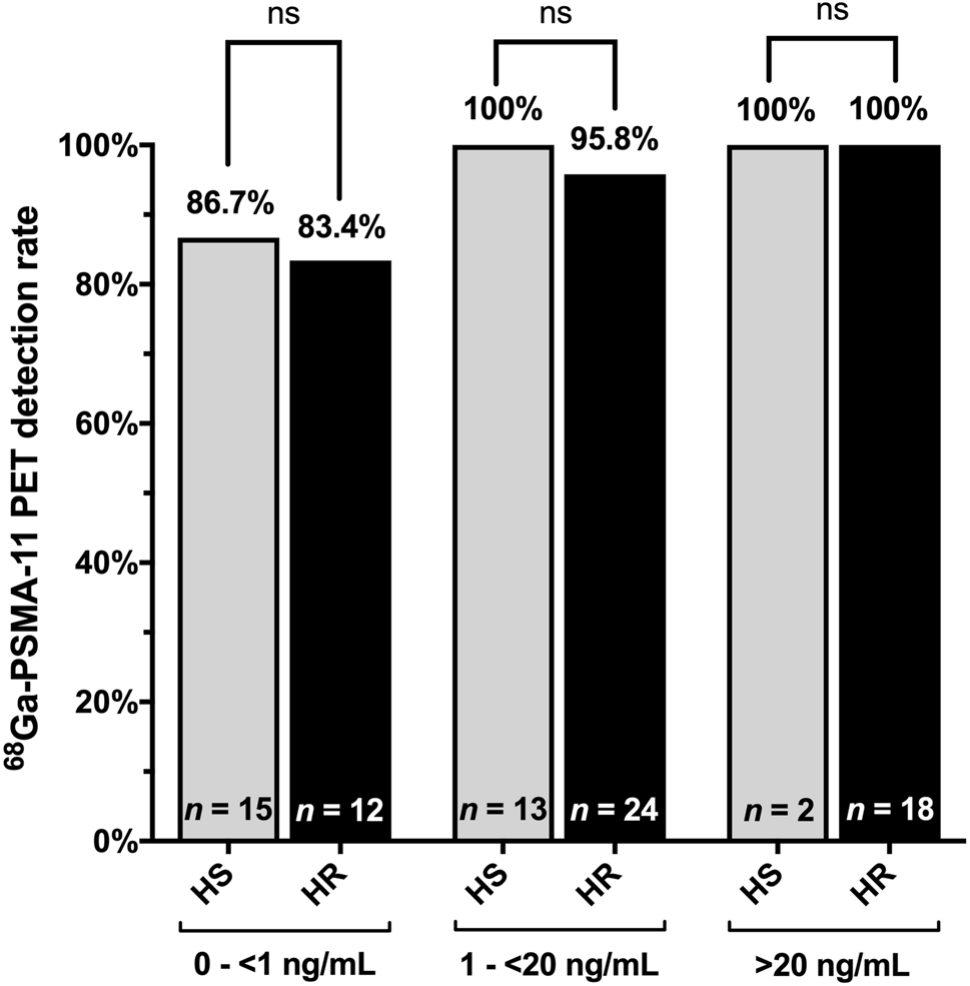
Comparison of ^68^Ga-PSMA-11 PET imaging detection rate between hormone-sensitive (HS) and hormone resistant (HR) PCa patients stratified by PSA subgroups at time of scan. Data is shown as percentage of events. No statistically significant difference was observed between HS and HR patients in all PSA subgroups (all *p* > 0.05). A Fisher’s exact test was calculated; *p* values < 0.05 were considered statistically significant

Comparing the detection of PSMA-positive lesions between hormone-sensitive and hormone-resistant PCa patients showed no statistically significant difference (all *p* > 0.05). In the subgroup with PSA values 0 to < 1 ng/mL PSMA-positive lesions were found in 13 of 15 (86.7%) patients with hormone-sensitive PCa, and in 10 of 12 (83.4%) patients with hormone-resistant PCa. In the subgroup of patients with PSA values 1 to < 20 ng/mL and > 20 ng/mL corresponding detection rates were 100% (13 of 13 patients) vs. 95.8% (23 of 24 patients) and 100% (2 of 2 patients) vs. 100% (18 of patients), respectively. (Fig. 3).

### Evaluation of oligometastatic patients with ≤ 3 PSMA-positive lesions

**Fig. 4:**
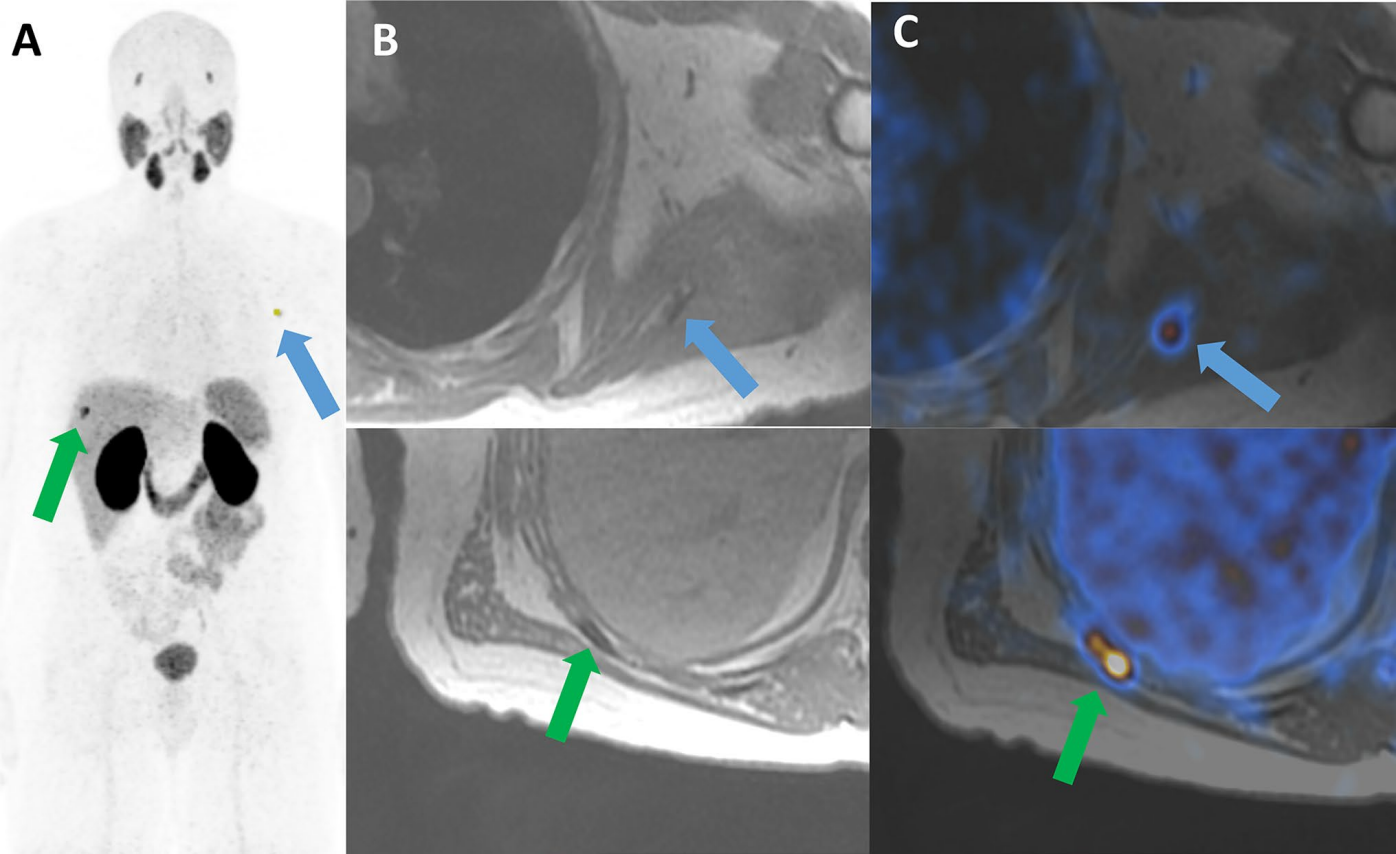
Patient with oligometastatic prostate cancer. 71-year-old patient initially following laparoscopic prostatectomy for pT3b pN0 cM0, Gleason 4 + 5 PCa. Restaging using ^68^Ga-PSMA-11 PET/MRI is performed due to a rising PSA to 0.8 ng/ml under established ADT using Leuporelin. **a** Coronal MIP of ^68^Ga-PSMA-11 with corresponding axial slides of fat weighted LAVA-DIXON slides **b** over the left scapular (blue arrow) and right rip (green arrow) metastases, and fused PET/MR **c** images showing the intense uptake in both lesions

Of 84 patients 19 (22.6%) were classified as having oligometastatic disease (Table 2). All patients had metachronous oligometastatic disease, no patient had lymph node only disease. Median time since the initial PCa treatment was 42.5 months. In this subgroup 47.4% (*n* = 9/19) had a PSA value 0 to < 1 ng/mL at scan time, 47.4% (*n* = 9/19) 1 to < 20 ng/mL and 5.3% (*n* = 1/19) > 20 ng/mL. In total, 52.6% (*n* = 10) were hormone-resistant and 47.4% (*n* = 9) hormone-sensitive. Median time since start of ADT was 25 months, in 11 patients (57.9%) < 50 months, in 6 (31.6%) 50–100 months and in 2 (10.5%) > 100 months. Almost all patients, 94.7% (*n* = 18) would theoretically have been eligible for MDRT in a personalized treatment setting (see Fig. 4). One patient had already undergone radiotherapy to the side of recurrence.

**Table 2:** Oligo-metastatic patients evaluated for metastasis-directed radiotherapy

Nr	vPSA (ng/ml)	sPSA (ng/ml)	HS/HR	nrMET	dADT (days)	LR	SUV_max_	LN	SUV_max_	LN Size (mm)	LN location	BM	SUV_max_	Activity (MBq)
1	20.9	0.001	HR	1	153	−		−				+	10.4	101
2	20.9	0.03	HR	2	144	−		−				+	13.4	134
3	8.98	0.03	HS	3	12	−		+	76.8	5	2	−		121
4	8.98	0.04	HS	2	18	−		+	6.3	4	2	−		116
5	2482	0.1	HR	3	85	+	5.1	+	13.2	8	1	+	6.4	139
6	4.5	0.27	HR	2	17	−		−				+	5.6	121
7	4.01	0.28	HS	1	38	+	6.5	−				−		112
8	19	0.8	HS	3	17	−		−				+	19	131
9	15.1	0.98	HR	1	54	+	15	−				−		148
10	28	1.72	HS	2	7	+	12.9	−				+	4.1	126
11	10.3	3.8	HR	3	67	−		−				+	20.5	171
12	109.6	3.8	HS	3	5	+	8	−				+	8.3	142
13	109.6	3.8	HS	1	10	+	7.7	−				−		151
14		5	HR	2	50	−		−				+	29.3	110
15		5	HR	2	64	−		−				+	31.6	146
16	8.6	5.9	HR	2	83	−		+	29.3	15	2	−		147
17	10.4	6.74	HS	1	40	−		−				+	21	149
18	202	15.3	HR	2	14	+	3.6	−				+	3.6	146
19	411	20	HS	2	11	+	28	+	5.8	6	1	−		140

LN location: 1 = pelvic, 2 = paraaortal

*vPSA* virgin prostate-specific antigen (at time of diagnosis), *sPSA* prostate-specific antigen at time of scan, *HS* hormone-sensitive, *HR* hormone-resistant, *nrMET* number of metastases, *dADT* duration of androgen deprivation therapy, *LR* local recurrence, *LN* lymph node metastasis, *LN* lymph node, *BM* bone metastasis, *SUV*_*max*_ maximum standardized uptake value

## Discussion

In this retrospective study analyzing the detection rate of ^68^Ga-PSMA-11 PET/CT or PET/MR in patients with advanced PCa and ongoing ADT we found a high overall detection rate of 94.0%. In each of the three PSA subgroups a detection rate of 85.2% (0 to < 1 ng/mL, *n* = 27), 97.3% (1 to < 20 ng/mL, *n* = 37) and 100% (> 20 ng/mL, *n* = 20) was observed, respectively.

Our results are in line with recently published data including a cohort of 30 patients with hormone-resistant PCa classified as non-metastatic by conventional imaging, in which ^68^Ga-PSMA-11 PET/CT revealed PSMA-positive disease in 90% of all patients [8]. The largest published cohort of 200 retrospectively analyzed patients with non-metastatic hormone-resistant PCa and a PSA serum level > 2 ng/ml by Fendler et al. reported a detection rate of 98%, and 55% of the patients with M1 disease [20]. These detection rates are considerably higher compared to pooled detection rates of 45% and 59% in hormone-naïve patients with biochemical recurrence following primary treatment and PSA values of 0.2–0.49 ng/ml and 0.5–0.99 ng/ml, respectively, reported by a recent meta-analysis [21]. Already in 2016, Pyka et al. compared ^68^Ga-PSMA-11 PET imaging with conventional bone scans in a cohort of 126 patients, including a subgroup of 40 patients with metastatic hormone-resistant PCa [22]. In these patients, no differences between the two modalities were observed on a patient basis even though, ^68^Ga-PSMA-11 PET imaging performed better in the identification of affected bone regions. However, mean PSA value in the mentioned subgroup was 446 ng/ml (range 0.97–3333 ng/ml). The high PSA value at scan might explain the comparable performance of both imaging methods.

Apart from overall detection rates, it remains unknown if ongoing ADT influences the PSMA positivity of ^68^Ga-PSMA-11 PET imaging. Preclinical research suggests an influence of short-term ADT on PSMA expression in PCa cells [23, 24] and multivariate analyses including larger patient cohorts revealed a positive correlation between the detection rate of ^68^Ga-PSMA-11 PET/CT with both PSA level and ongoing ADT at scan [25]. Already in 2017, a first case report showed an increased PSMA tracer uptake following short-term ADT in a patient with hormone-sensitive PCa. In addition, it raised the number of lesions visualized [6]. Recent data, including small patient cohorts with advanced hormone-resistant PCa, reported a 42–50% increased PSMA expression following treatment with either Enzalutamide or Abiraterone [7, 26]. In contrast, no increased PSMA expression was found in a study of 26 metastatic hormone-resistant PCa patients after a median of 3 months under Enzalutamide or Abiraterone [27] and a decreased PSMA expression was reported in a retrospective analysis of 10 hormone-resistant patients following start of ADT [28]. To minimize ADT as confounding factor, we included only patients with ongoing ADT in the present analysis. No significant differences in the detection rate of PSMA-positive lesions were observed between patients with hormone-sensitive and hormone-resistant PCa.

In addition, improved detection of small metastases using ^68^Ga-PSMA-11 PET imaging has led to a more frequent diagnosis of oligometastatic PCa. In the absence of randomized phase III trials, early clinical data reported an improved progression-free survival or overall survival when MDRT was added to standard systemic therapy in patients with oligometastatic disease [29]. In 2018, Ost et al. compared MDRT with surveillance in a retrospective cohort of 62 patients with oligorecurrent PCa [12]. The primary end point was ADT-free survival. ADT was started at symptomatic progression, progression to more than three metastases, or local progression of known metastases. Observed ADT-free survival was longer in patients treated with MDRT compared to those with surveillance alone. However, oligometastatic PCa was detected by choline PET imaging and not PSMA PET imaging. This might have influenced the analyzed cohort. A larger retrospective multicenter study including 305 patients with biochemical recurrent PCa found that concurrent ADT with MDRT significantly improved biochemical progression-free survival in patients with oligometastatic PCa detected by PSMA PET imaging [30]. In addition, an ongoing prospective phase II trial (NCT 04222634) aims to include patients with oligoprogressive metastatic hormone-resistant PCa who are treated with MDRT to all visible progressive lesions. However, progression in this study performed by Berghen et al. is based on conventional imaging and not ^68^Ga-PSMA-11 PET imaging [31]. In our cohort of 84 patients 19 (22.6%) were classified as having oligometastatic PCa. Half of these patients (52.6%) were classified as hormone-resistant, while 47.4% were classified as hormone-sensitive. Almost all patients, 94.7% (*n* = 18) would theoretically have been eligible for MDRT. However, different definitions of oligometastatic PCa using different imaging modalities hinder a direct comparison of published results. Future prospective trials need to address the question, whether a metastasis-directed therapy can improve overall survival in a sub-group of patients with oligometastatic PCa diagnosed by ^68^Ga-PSMA-11 PET imaging [32]. A limitation of our study is its retrospective nature leading to an inherent selection bias. Due to retrospective data acquisition there is a lack of clinical information in some patients. Furthermore, the reported patient collective is still relatively small and lacks histopathological confirmation of PSMA-positive lesions, given that biopsy is frequently omitted in clinical practice for patients with multiple lesions in advanced disease.

## Conclusion

In this study, we observed an overall very high detection rate of 94% using PSMA PET imaging in patients with advanced PCa and ongoing ADT. Even in a majority of patients with very low PSA values < 1 ng/ml at the time of scan PSMA-positive lesions were found.
